# *Leptospira interrogans* biofilm formation in *Rattus norvegicus* (Norway rats) natural reservoirs

**DOI:** 10.1371/journal.pntd.0009736

**Published:** 2021-09-08

**Authors:** Ana Amélia Nunes Santos, Priscyla dos Santos Ribeiro, Geórgia Virgínia da França, Fábio Neves Souza, Eduardo Antônio Gonçalves Ramos, Cláudio Pereira Figueira, Mitermayer G. Reis, Federico Costa, Paula Ristow

**Affiliations:** 1 Institute of Biology, Federal University of Bahia, Salvador, Brazil; 2 Institute of Biological Science, Federal University of Minas Gerais, Belo Horizonte, Brazil; 3 Institute of Collective Health, Federal University of Bahia, Salvador, Brazil; 4 Gonçalo Moniz Institute, Oswaldo Cruz Foundation, Ministry of Health, Salvador, Brazil; 5 Faculty of Medicine of Bahia, Federal University of Bahia, Salvador, Brazil; 6 Department of Epidemiology of Microbial Diseases, School of Public Health, Yale University, New Haven, Connecticut, United States of America; Rocky Mountain Laboratories, NIAID, NIH, UNITED STATES

## Abstract

*Rattus norvegicus* (Norway rat) is the main reservoir host of pathogenic *Leptospira*, the causative agent of leptospirosis, in urban environments. Pathogenic *Leptospira* forms biofilms in the environment, possibly contributing for bacterial survival and maintenance. Nonetheless, biofilms have not yet been studied in natural animal reservoirs presenting leptospiral renal carriage. Here, we described biofilm formation by pathogenic *Leptospira* inside the renal tubules of *R*. *norvegicus* naturally infected and captured in an urban slum endemic for leptospirosis. From the 65 rats carrying *Leptospira* in their kidneys, 24 (37%) presented biofilms inside the renal tubules. The intensity of leptospiral colonization in the renal tubules (OR: 1.00; 95% CI 1.05–1.1) and the type of occlusion pattern of the colonized renal tubules (OR: 3.46; 95% CI 1.20–9.98) were independently associated with the presence of *Leptospira* biofilm. Our data showed that *Leptospira interrogans* produce biofilms during renal chronic colonization in rat reservoirs, suggesting a possible role for leptospiral biofilms in the pathogenesis of leptospirosis and bacterial carriage in host reservoirs.

## Author summary

Leptospirosis is an infectious disease caused by pathogenic *Leptospira* bacteria. The main reservoir hosts of *Leptospira* are the brown rats (*Rattus norvegicus*), which are chronically colonized in the kidneys. Leptospires form biofilms, which are communities of microorganisms embedded in an extracellular polysaccharidic matrix. *Leptospira* pathogenesis in reservoir hosts is poorly understood. We captured 87 brown rats from an impoverished urban community that is endemic for leptospirosis. To investigate the biofilm in the rats’ kidneys, we co-localized leptospires and saccharides of the biofilm extracellular matrix in the renal tubules, using immunohistochemistry anti-*Leptospira* and carbohydrate staining, respectively. We quantified *Leptospira* using molecular tools and characterized the biofilm using electron microscopy. We analysed demographic data to identify variables correlated with renal carriage. We found that *Leptospira* infected 78 rats. From those, 65 were positive for immunohistochemistry in the kidneys and 24 (37%) were biofilm-positive. We found significant positive correlation between the intensity of colonization and the presence of biofilm in the kidneys. The intensity of colonization was also associated with the rats’ gender and age. Biofilm formation by *Leptospira* in the kidneys of natural reservoir rats fills a gap into the knowledge of leptospirosis pathogenesis.

## Introduction

Leptospirosis is an infectious disease of public health global importance [1]. It is estimated that more than one million cases and nearly 60 thousand deaths occur yearly in the world due to leptospirosis [2]. The incidence of this zoonotic disease is higher in developing and tropical countries, especially during rainy seasons [3,4]. Leptospirosis is caused by pathogenic spirochetes from the genus *Leptospira*, which is composed of 66 pathogenic and saprophytic species [5,6]. The development of leptospirosis depends, among others, on epidemiologic factors such as sanitation and flooding, on the pathogenicity and infecting dose of *Leptospira*, and on host susceptibility to infection [7].

*Rattus norvegicus* (Norway rats) are the main reservoir hosts of leptospires in urban settings worldwide. It is estimated that naturally infected Norway rats daily excrete approximately 9.0e+10 leptospires in the urine, strongly contaminating the environment [8]. Pathogenic *Leptospira* densely colonize the kidneys of rat reservoir hosts, where they form aggregates in the proximal renal tubules [9,10]. Experimentally infected *R*. *norvegicus* with a high dose of pathogenic leptospires remain asymptomatic and with stable renal colonization, what is observed at the first week post infection, persevering for more than four months [11]. Histopathological analysis of the kidneys of naturally infected *R*. *norvegicus* revealed minor alterations as interstitial nephritis and tubular epithelial hyaline droplets. However, it was not possible to detect if those alterations were related to *Leptospira* infection, once numerous environmental exposures could cause similar alterations [9,12]. During infection in chronic animal models, leptospires form biofilm-like structures during renal tubular colonization [13], what may explain the antibiotic tolerance observed when the treatment occurs during the disease chronic phase [14].

Bacteria of the genus *Leptospira* form biofilms *in vitro* [15] and in rural environment [16]. Biofilms are communities of microorganisms attached to a surface and involved by a self-produced exopolymeric matrix [17,18]. The transcriptome of saprophytic *Leptospira biflexa* revealed important insights into *Leptospira* biology during biofilm formation. Of note, there was a great shift in gene regulation during biofilm, as the diminishment of alginate gene expression, upregulation of a putative gene for a virulence-associated protein, and upregulation of outer membrane proteins (OMPs) encoding genes [19]. A recent study analyzed *in vitro* several aspects of the biofilm formed by pathogenic *Leptospira* and showed that inside the biofilm leptospires are protected against stressful environmental conditions as pH and temperature [20].

The biofilm lifestyle is ubiquitous in Bacteria. This ubiquity is related to the protection offered by biofilms against harsh environmental and host conditions [18,21]. Biofilms are described as colonization and virulence factors [22], participating in the pathogenesis of several diseases caused by pathogenic or opportunistic bacteria [23,24]. Bacterial biofilms are associated with many medical chronic conditions as cystic fibrosis pneumonia, dental plaque, and catheter contamination, among others [24–27]. The spirochete *Borrelia* form biofilms *in vitro* and *in vivo* in the skin of infected patients diagnosed with borrelial lymphocytoma [28,29]. Biofilms were also described as crucial for the transmission of *Yersinia pestis* and host-pathogen interactions in plague, due to biofilm formation in the midgut of fleas [30].

Knowledge about pathogenic *Leptospira* biology, host-pathogen interactions, and survival inside the hosts, as well as their transmission mechanisms to other hosts are of outermost importance to understand and counteract the infection in susceptible hosts [31,32]. In this study we demonstrated *Leptospira* biofilms in the kidneys of naturally infected *Rattus norvegicus* captured in an urban slum endemic for leptospirosis. We identified the factors associated with renal biofilm formation and characterized the histopathological kidney alterations in natural reservoir rats.

## Methods

### Ethics statement

This work was approved by the Institutional Animal Care and Use Committee at the Oswaldo Cruz Foundation (Salvador, Brazil; protocol number 003/2012).

### Study sites and animals

Animal captures followed a previously described methodology with few modifications (COSTA et al., 2015a). Briefly, from May 2013 to August 2014, we captured 86 Norway rats (*Rattus norvegicus*) in Pau da Lima slum, in Salvador, Bahia. Pau da Lima is an urban community with 0.46 Km^2^ area and four valleys [33], and was selected for this study given its high incidence of severe human leptospirosis [34]. We systematically sampled the study site by setting two Tomahawk live traps at each of 108 sampling points [35], and recorded the capture site and entered/validated demographic data in Redcap database. We euthanized the rats, recorded the site of collection, sex, and weight, and used mass/weight as a proxy for estimating rat’s age, dividing them into juveniles (≤ 200 g), sub-adults (201–400 g) and adults (≥ 401 g). We collected the rats’ urine directly from the bladder using a 1mL syringe and froze the urine in -80° C until qPCR analysis [8]. During necropsies, we collected and preserved the right kidney in 10% buffered formalin and further processed for histology. We also collected and divided the left kidney; half of it we preserved in -80°C [8] and the other half was processed for scanning electron microscopy (SEM), as described below.

### Quantitative real-time PCR (qPCR) of *Leptospira* load in kidneys

We performed quantitative real-time polymerase-chain reaction (qPCR) strictly as described by COSTA et al., 2015b. Briefly, we extracted DNA from 200 μL of urine and 25 mg of frozen kidney using Maxwell 16 System DNA Purification Kits (Promega Corp., USA). We performed qPCR for pathogenic leptospires using 5’ nuclease (Taq-Man) assay, and primers for *lipL32*, a gene solely present in pathogenic leptospires [36]. We performed the quantitative amplification using an ABI 7500 Real-Time PCR System (Applied Biosystems, USA).

### Histological processing, immunofluorescence (IF), and immunohistochemistry (IHC)

We screened the kidney sections for *L*. *interrogans* infection using immunofluorescence (IF) qualitative method imprint technique, following the protocol described by CHAGAS-JUNIOR et al. [37]. Next, we embedded the kidneys in paraffin wax, cut the blocks in 2-μm serial sections and processed for histopathology, in the following order: (1) hematoxylin-eosin (HE); (2) immunohistochemistry (IHC) anti-*Leptospira interrogans*; (3) periodic acid-Schiff stain (PAS); (4) PAS Silver Methenamine (PAS-M); (5) Alcian blue pH 2.5 (AB); (6) Mayer’s Mucicarmine (MM); (7) AZAN; (8) Picrosirius Red (PIFIG). We used HE, PAS, PAS-M, AZAN and PIFIG to analyze pathologic alterations, according to routine protocols. We then applied a questionnaire for histopathological analysis.

For biofilm detection, we used AB pH 2.5 and MM (special stains for carbohydrates), according to routine protocols. For MM staining, we deparaffinized the samples in xylol baths twice for 10 min each; immersed in absolute alcohol baths twice for 30 s each; hydrated in tap water; and placed the slides in Weigert’s iron hematoxylin for 7 min. Then, we washed the samples under tap water for 10 min; added mucicarmine solution for 1 h; washed quickly in non-pyrogenic distilled water, added metanil yellow for 1 min, and washed again in non-pyrogenic distilled water. We then dehydrated the samples in 95% alcohol; placed 2x in absolute alcohol baths for 30 s each; clarified in xylol baths twice for 30 s each; and assembled the slides. For AB staining, we deparaffinized the samples in xylol baths twice for 10 min each; immersed in absolute alcohol baths twice for 30 s each and hydrated under tap water. Then, we immersed the slides in a 3% acetic acid solution for 10 min, followed by immersion in 2% alcian blue solution for 30 min and washed in non-pyrogenic distilled water to remove the excess dye. We immersed the slides into Harris hematoxylin for 1 min, rinsed in tap water for 5 min, dehydrated in absolute alcohol baths three times for 3 min each, clarified in two xylol baths, and assembled the slides. We obtained the microscopy images using an Olympus BX51 optical microscope with objectives of 20x and 40x and the Image-Pro Plus software (Media Cybernetics, USA). We analyzed biofilm formation only in the kidneys of positive rats for IHC anti-*L*. *interrogans*.

We performed IHC analysis according to CRODA et al. (2008) [38], with the following modifications: we blocked the slides with 10% skimmed milk in 1× phosphate buffered saline (NaCl 140 mM; 2.7 mM KCl; 10 mM Na2HPO4; 1.8 mM KH2PO4; pH 7.4) and incubated with rabbit primary antibodies anti-*Leptospira interrogans* serovar Icterohaemorrhagiae strain RGA diluted 1:1.000 at room temperature for 1 h. As negative controls, we used kidneys of negative rats for *L*. *interrogans* infection. Once we identified renal tubules positive to IHC anti-*L*. *interrogans*, we performed co-localization of these tubules in serial renal sections with tubules concomitantly positive for AB staining.

### Patterns of leptospiral colonization in rat kidneys

We analyzed different patterns of leptospiral colonization in *R*. *norvegicus* kidneys using a previous methodology described by SANTOS et al., 2015. We inferred colonization intensity by counting the number of IHC positive colonized tubules (CTs) in a half-kidney cortex. This information generated the variable “number of colonized tubules”, in this work denominated CT count, considered to be a quantitative measurement of infection. We analyzed the distribution of CTs by performing a qualitative evaluation, registering if CTs were observed isolated in the cortex (one CT) or agglomerated (two or more CTs), and with focal or multifocal distribution. Finally, we qualitatively evaluated the tubular intraluminal IHC, considering two patterns: kidney tubules with IHC marking restricted to the renal epithelial membrane (considered as partial occlusion of the CT) or kidney tubules with IHC marking both restricted to the renal epithelial membrane and completely occluding the lumen of CT (considered as partial/complete occlusion of the CT).

### Electron microscopy using ruthenium red probe

Using a sterile scalpel, we cut fresh half kidneys of captured rats in 1–2 mm cubes, fixed in 2% glutaraldehyde/0.1 M sodium cacodylate, and kept at 4°C until processing. A non-colonized rat kidney was processed as negative control. We then transferred the samples to new tubes containing the same fixative with or without 0.2% ruthenium red (RR), washed in 0.1 M sodium cacodylate and post-fixed in 1% osmium tetroxide/0.2 M sodium cacodylate for one hour. We washed and dehydrated the samples in graded series of ethanol (from 30–100%), followed by critical point dry (Leica EM CPD030, Austria), sputter-coated with gold (DESCK IV; Denton Vacuum, USA), and examined using a scanning electron microscope (JSM6394LV; JEOL, Japan) operated at 10 kV. For this analysis, we used a subsampling of four positive rats for *Leptospira* infection with renal biofilm and one negative rat as a control.

### Statistical analysis

We considered the rats positive to renal infection when they were positive for IF and/or IHC and/or qPCR anti-*Leptospira*. Rats were considered to have biofilms when they were positive in the co-localization technique (AB and IHC anti-*Leptospira*). Sixty-seven rats were analyzed for patterns of leptospiral colonization and 64 rats with positive IHC anti-*L*. *interrogans* were analyzed for biofilm presence. We assessed the association of *Leptospira* infection, biofilm formation and patterns of renal tubules colonization with demographic data (sex, weight category and site of collection) and *Leptospira* load in rats’ urine. We transformed leptospiral load (GEq of leptospiral DNA/mL) into log for all the analyses. We used Chi-square test to investigate *Leptospira* infection and biofilm formation occurrence in the rats’ population, and Kruskal-Wallis to evaluate leptospiral load in urine and CT count. To evaluate *Leptospira* infection and renal biofilm association with demographic characteristics and renal tubules pattern of colonization, we used Pearson Chi-square test or Fisher’s Exact test (n < 5). We used generalized linear model (GLM) to analyze the risk factors associated with *Leptospira* infection occurrence and renal biofilm formation in the chronically infected host, *R*. *norvegicus*. GLM was also used to analyze demographic data associated with histological characteristics of infected rats. We used R Version 1.3.1093 [39] and considered differences with p<0.05 as significant. All results are available in S1 Table.

## Results

### Reservoir *Rattus norvegicus* present renal biofilm formed by pathogenic *Leptospira*

**Fig 1:**
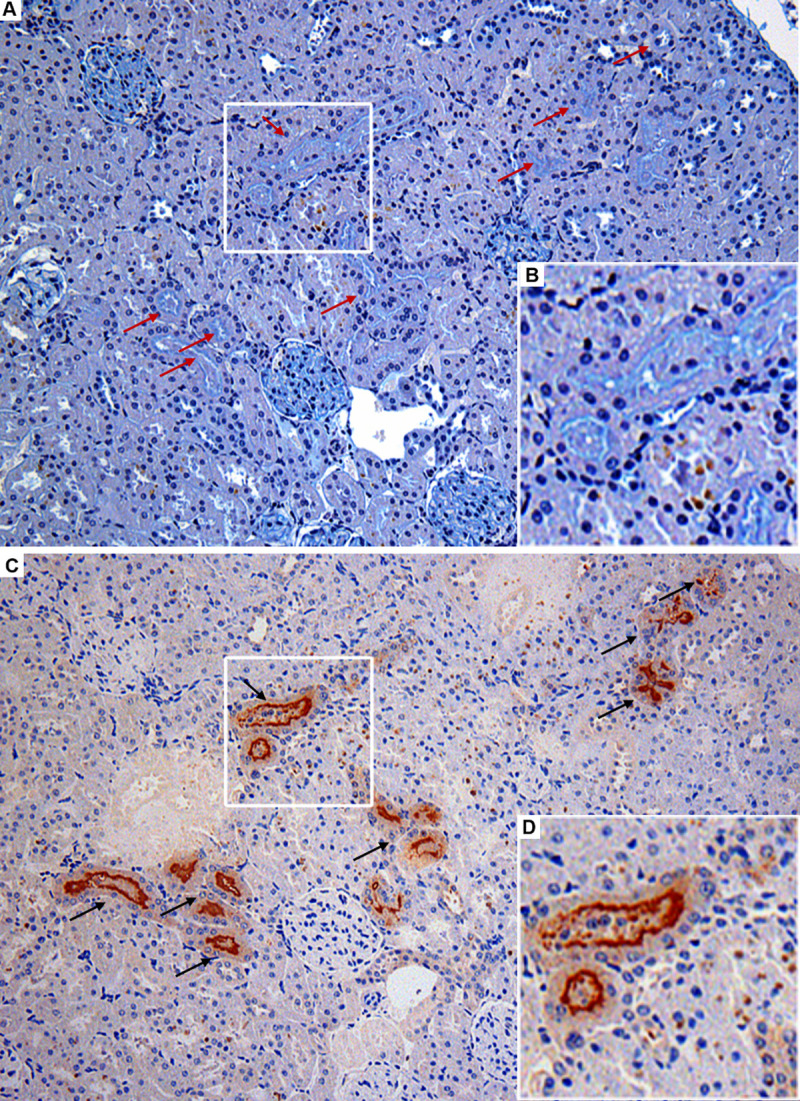
Histopathological investigation of leptospiral infection and biofilm formation in kidney serial sections of wild *Rattus norvegicus* naturally infected with pathogenic *Leptospira interrogans*. (A) Alcian Blue (AB) positively stained renal tubules (red arrows) observed in light turquoise blue, indicating the presence of biofilm matrix; insert B with detail of biofilm staining. (C) Renal tubules positive for IHC anti-*L*. *interrogans* demonstrating leptospiral colonization of proximal tubules (black arrows); insert D with detail of colonized tubule. Note that the serial sections showed in A and D are from the same region of one rat kidney, evidencing the co-localization of tubules concomitantly positive in AB (red arrows) and IHC (black arrows). Magnification, x 200.

We captured 87 *R*. *norvegicus* from May 2013 to August 2014, at Pau da Lima neighborhood (S1 Fig). From those, 78 (90%) rats were positive for *Leptospira* infection (p = 1.38–13) using one of the methods previously described. From the 78 rats, 65 were positive for immunohistochemistry (IHC) anti-*Leptospira* and were analyzed for the presence of renal biofilm. Twenty-four (37%) rats were positive (p = 0.04) (Table 1) for renal biofilm according to co-localization of Alcian Blue (AB) staining (Fig 1A and 1B) and IHC anti-*Leptospira* (Fig 1C and 1D), confirming the presence of polysaccharidic matrix and *Leptospira* biofilms inside the proximal renal tubules of infected rats. None of the kidneys were positive for Mayer mucicarmine (MM) staining (S2 Table). Negative controls did not stain for either AB or MM (S2 Table). We used as positive controls standard sections of dog intestine, which were positive for both AB and MM (S2 Table).

**Table 1:** Demographic characteristics, patterns of renal colonization and *Leptospira* shedding in wild *Rattus norvegicus* (Norway rats) positive for *Leptospira* infection and renal biofilm.

Characteristics	*Leptospira* positive rats No. ^¥^	*Leptospira* biofilm positive rats No. (%^⁂^)	OR (CI)
**Total**	65	24 (37) *	
**Sex**			
*Intercept*			0.57 (0.29–1.12)
Female	36	13 (36)	*Ref*.
Male	29	11 (38)	1.08 (0.39–2.98)
**Weight categories**			
*Intercept*			0.40 (0.13–1.28)
Adult	14	4 (29) *	*Ref*.
Sub-adult	38	15 (39)	1.56 (0.31–7.82)
Juvenile	13	5 (38)	1.63 (0.43–6.16)
**Site of capture**			
*Intercept*			0.22 (0.05–1.03)
Valley 1	11	2 (18) *	*Ref*.
Valley 2	6	1 (17)	0.90 (0.06–12.58)
Valley 4	33	17 (52)	4.78 (0.89–25.59)
**Leptospiral shedding in urine**			
*Intercept*			0.14 (0.01–1.29)
Mean log_10_ qPCR urine^1^	NA	6.34 *	1.24 (0.94–1.63)
**CT count**			
*Intercept*			**0.28 (0.13–0.61)**
Mean of CT^2^	NA	229	**0.99 (1.0–1.01)** **
**CT occlusion pattern**			
*Intercept*			**0.32 (0.15–0.68)**
Partial	37	9 (24) *	*Ref*.
Partial/complete	28	15 (53)	**3.59 (1.25–10.32)** **
**CT distribution**			
*Intercept*			0.62 (0.36–1.06)
No. agglomerated CT	55	21 (39)	*Ref*.
No. isolated CT	10	3 (30)	0.69 (0.16–2.98)

* Significant differences in descriptive statistical analysis (P<0.05);

** Bold items indicating significant OR in generalized linear model analysis (P<0.05);

^¥^ Sample with 65 animals positive for IHC anti-*Leptospira* and analyzed for the presence of renal biofilm.

^⁂^ Percentage relative to the number of *Leptospira* positive rats;

^**1**^The mean log_10_ qPCR urine of negative rats for renal biofilm was 7.34.

^**2**^The mean of CT count of negative rats for renal biofilm was 113. *Ref*.: reference category; refers to the default chosen category against which other categories are compared to in regression models when we use categorical dependent variables. *Intercept*: regression constant; predicts a linear value when all predictor variables are set to zero. **Abbreviation:** OR, Odds Ratios; CI, Confidence Interval.

**Table 2:** Histopathological alterations observed in chronically infected *Rattus norvegicus* with the presence of renal biofilm.

Alteration	Renal biofilm positive samples No. (%)
**Total**	**24**
Mesangial hypercellularity	2 (8)
Mesangial matrix hyperplasia	5 (21)
Hyaline-goticular degeneration	12 (50)
Cylinders	3 (13)
Epithelial tubular regeneration	1 (4)
Minimal glomerular alterations	3 (13)
Focal proliferative glomerulonephritis	0
Focal mesangial proliferative glomerulonephritis	0
Focal mesangial segmental proliferative glomerulonephritis	1 (4)
Acute tubular necrosis	0
Moderate chronic interstitial nephritis	4 (17)
Discrete chronic interstitial nephritis	7 (29)
Kidney within normal parameters	4 (17)

The population of rats with renal biofilm was homogeneously comprised of 11 males (46%) and 13 females (54%), not statistically significant; 17 (71%) were collected at valley 4 (p = 4.2e-06); and the majority 15 (63%) were sub-adults (p = 9.6e-05) (Table 1). We observed that the mean of leptospires’ shedding in urine was lower in rats with renal biofilm (n = 24; 2.23e+06 GEq), compared with infected rats with no biofilm (n = 41; 2.2e+07 GEq) (p = 2.2e-10) (Table 1). However, generalized linear model (GLM) analysis did not show statistical association between renal biofilm formation and demographic characteristics and leptospiral urine shedding.

We analyzed the association of renal biofilm formation with renal colonization patterns. We observed an average of 229 colonized tubules (CT) count in rats with renal biofilm, while 113 in rats infected but negative for biofilm (p = 0,25) (Table 1). The majority of rats presenting renal biofilm (15; 63%) presented IHC anti-*L*. *interrogans* marking pattern of partial to complete occlusion of CTs (p = 0,037) and 21 rats (88%) presented agglomerated CT distribution (p = 0.73) (Table 1). GLM showed that the intensity of colonized tubules increased the chance of renal biofilm formation (Table 1). Furthermore, the partial to complete pattern of tubule colonization increased in more than three times the chance of having renal biofilm (Table 1).

### Biofilm matrix is labeled by ruthenium red in scanning electron microscopy

**Fig 2:**
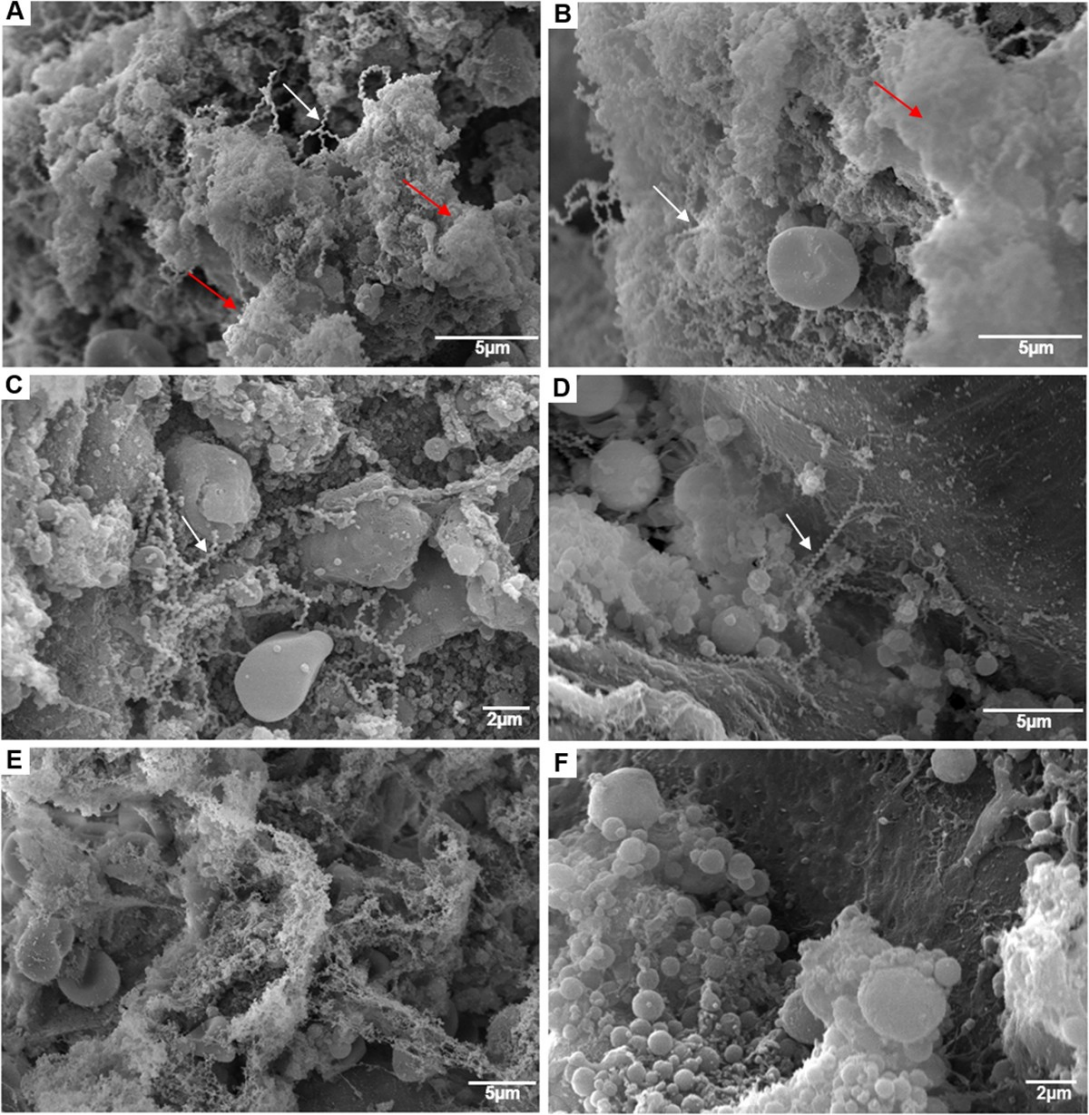
Scanning electron microscopy (SEM) of leptospiral renal biofilm and its matrix in wild naturally colonized *Rattus norvegicus*. A and B–SEM of colonized kidney with ruthenium red (RR) showed *Leptospira a*gglomerates (white arrows) surrounded by an anionic exopolysaccharidic matrix (red arrows) inside the renal tubules. C and D–SEM of colonized kidney without RR, where leptospires are evidenced agglomerated (C) or isolated (D), without the presence of the matrix. E–SEM using RR of *R*. *norvegicus* negative control. F–SEM without RR of *R*. *norvegicus* negative control.

We stained chronically infected rats’ kidneys with ruthenium red (RR) and analyzed by Scanning Electron Microscopy (SEM). We observed dense leptospiral colonization, forming aggregates inside the renal tubules (Fig 2A and 2B–white arrows), with heavy deposition of amorphous extracellular matrix between leptospires, often covering and embedding spirochete bacteria, compatible with biofilm morphology (Fig 2A and 2B–red arrows). When we analyzed the kidneys of infected rats without RR staining, we observed the presence of isolated and agglomerated leptospires inside the renal tubules, without amorphous extracellular matrix in-between and embedded-in (Fig 2C and 2D–white arrows). Finally, we did not observe biofilm nor *Leptospira* in negative controls (Fig 2E and 2F).

### Kidneys’ histopathological analyses of *R*. *norvegicus* positive for renal biofilm

Histopathological analysis revealed minimal alterations in the kidneys of rats with renal biofilm (Table 2). We observed the occurrence of hyaline-goticular degeneration (n = 12; 50%), mesangial matrix hyperplasia (n = 5; 21%); mesangial hypercellularity (n = 2; 8%), and epithelial tubular regeneration (n = 1; 4%) (Table 2).

### Demographic data and leptospiral shedding in rat’s urine are associated with CT count

**Fig 3:**
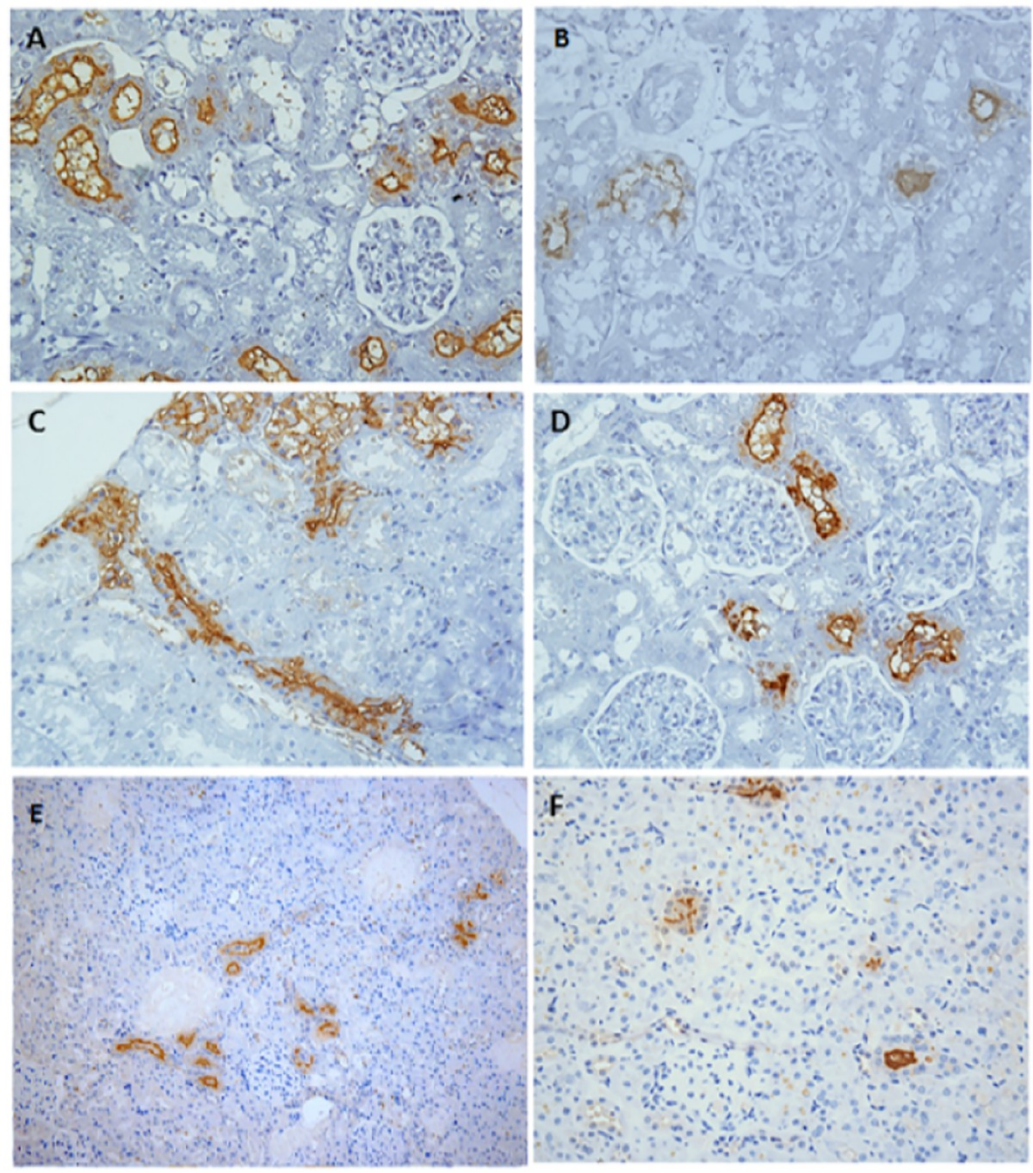
Patterns of kidney colonization marking of naturally infected *Rattus norvegicus*. Immunohistochemical representative images of kidney with (A) high intensity of colonized tubules (CTs); (B) low intensity of CTs; (C) partial to complete pattern of CT lumen occlusion; (D) marking restricted to the membrane of renal epithelium (partial occlusion); (E) agglomerated CTs distributed in the renal cortex; (F) isolated CTs distributed in the cortex. A, B, C and D: magnification, x 400. E and F: magnification, x 200.

We observed a homogeneous distribution of infected rats between males (n = 38) and females (n = 40), although uneven among negative rats (8 females and 1 male) (p = 0.03) (Table 3). Infected rats were mainly sub-adult (n = 43, 55%) (p-value = 0.006), collected at valley 4 (n = 44, 56%) (p = 0.24), and presented mean of leptospiral shedding of 1.45e+07 GEq (Table 3). For the characterization of leptospiral colonization patterns, we analyzed 68 rats, which were positive for IHC and/or IF anti-*Leptospira* (Fig 3). There was a range from 1 to 612 CT count, with an average of 154 CT count (Table 3). Twenty-eight rats (41%) presented IHC *anti-L*. *interrogans* marking partial to complete pattern of CT occlusion (Table 3 and Fig 3C), whilst 40 rats (59%) presented marking restricted to the membrane of renal epithelial cells, characterizing partial occlusion of CTs (Table 3 and Fig 3D). Ten rats (15%) presented an isolated distribution pattern (Table 3 and Fig 3E), whereas 58 (85%) were agglomerated (Table 3 and Fig 3F).

**Table 3:** Demographic characteristics, patterns of renal colonization and *Leptospira* shedding in wild *Rattus norvegicus* (Norway rat) naturally infected.

	*Leptospira* infection			CT count^¥^		CT distribution (n = 68)^¥^			CT occlusion pattern (n = 68) ^¥^		
*Predictors*	Rats No.	Positive No. (%^⁂^)	OR (CI)	Mean	*OR (CI)*	A. No. (%)	I. No. (%)	*OR (CI)*	P. No. (%)	P/C No. (%)	*OR (CI)*
**Total**	87	78 (90)*		154*		58 (85)	10 (15)		40 (59)	28 (41)	
**Sex categories**											
*Intercept*			5.0 (2.3–10.7)		146.2 (142.3–150.1)			0.12 (0.04–0.34)			0.54 (0.28–1.06)
Female	48	40 (83)*	*Ref*.	146	*Ref*.	33 (57)	4 (40)	*Ref*.	24 (60)	13 (46)	*Ref*.
Male	39	38 (97)	7.6 (0.9–63.6)	164	1.12** (1.08–1.16)	25 (43)	6 (60)	1.98 (0.50–7.77)	16 (40)	15 (54)	1.66 (0.65–4.59)
**Weight categories**											
*Intercept*			1,2E+8 (0–Inf)		174.4 (167.8–181.2)			0.07 (0.01–0.54)			0.87 (0.32–2.41)
Adult	15	15 (100)*	*Ref*.	174	*Ref*.	14 (24)	1 (10)	*Ref*.	8 (20)	7 (25)	*Ref*.
Juvenile	27	20 (74)	0.00 (0–Inf)	207	1.18** (1.12–1.25)	10 (17)	3 (30)	4.20 (0.38–46.49)	8 (20)	5 (18)	0.71 (0.16–3.23)
Sub-adult	45	43 (95)	0.00 (0–Inf)	130	0.74** (0.71–0.78)	34 (59)	6 (60)	2.47 (0.27–22.44)	24 (60)	16 (57)	0.76 (0.23–2.52)
**Site of collection**											
*Intercept*			1,2E+8 (0–Inf)		175.1 (167.4–183.1)			0.10 (0.01–0.78)			0.83 (0.25–2.73)
Valley 1	11	11 (100)	*Ref*.	175	*Ref*.	10 (17)	1 (10)	*Ref*.	6 (15)	5 (18)	*Ref*.
Valley 2	8	6 (75)	0.00 (0–Inf)	162	0.93 (0.86–1.00)	5 (9)	1 (10)	2.0 (0.1–39.1)	4 (10)	2 (7)	0.60 (0.08–4.76)
Valley 4	50	44 (88)	0.00 (0–Inf)	146	0.83** (0.79–0.88)	31 (53)	5 (50)	1.61 (0.17–15.5)	21 (52)	15 (54)	0.86 (0.2–3.3)
**Leptospiral shedding in urine**											
*Intercept*					81.5 (75.4–88.1)			0.70 (0.1–4.0)			0.26 (0.07–1.0)
Mean qPCR urine		7.16			1.09** (1.08–1.10**)**	5.2	4.21	0.77 (0.6–1.1)	3.8	5.4	1.1 (0.94–1.28)

*Results statistically different (p-value < 0.05);

** Bold items indicating significant OR in GLM analysis (P<0.05);

^⁂^ Percentage relative to the number rats;

^**¥**^Data referring to histopathological analysis of 68 positives rats for IHQ and/or IF anti-*Leptospira*. *Ref*.: reference category; refers to the default chosen category against which other categories are compared to in regression models when we use categorical dependent variables. *Intercept*: regression constant; predicts a linear value when all predictor variables are set to zero.

**Abbreviations:** OR, Odds Ratios; CI, Confidence intervals; A., Agglomerated; I., Isolated; P., Partial; P/C, Partial to complete.

We used generalized linear models to analyze leptospiral renal colonization patterns associations with demographic data and intensity of leptospiral shedding in the rats’ urine. The intensity of colonized tubules was associated with demographic data and leptospiral shedding in urine. Male and juvenile rats had a higher chance of having more CT count, while rats captured at valley 4 were more likely to have fewer (Table 3). Finally, the greater the intensity of leptospiral shedding in the rats’ urine, the greater is the chance of having more CT count (Table 3).

## Discussion

We identified and characterized leptospiral biofilm formation inside the renal tubules of *R*. *norvegicus* naturally infected with pathogenic *Leptospira*. By analyzing demographic data and histological patterns of renal colonization by leptospires, we identified the risk factors associated with biofilm formation. Additionally, we investigated histopathological alterations in the rats’ kidneys positives for biofilm. Finally, we characterized *R*. *norvegicus* infection by pathogenic *Leptospira*, analyzing demographic data, leptospiral shedding intensity in urine, and histological patterns of colonization.

Reservoir hosts infected with *Leptospira* and experimentally infected rats (chronic model of disease) present dense renal tubules colonization [9,13]. In the present study, from the 65 infected Norway rats analyzed, more than a third (37%) had pathogenic *Leptospira* forming biofilms inside renal tubules. Biofilm formation by pathogenic bacteria has been described in other hosts. *Borrelia burgdorferi*, the causative agent of Lyme disease, are spirochetes capable of forming biofilms *in vitro*, in the midguts of infected ticks, and in the skin tissue of borrelial lymphocytoma patients [28,29,40]. *Yersinia pestis*, the causative agent of bubonic plague, form biofilm inside the flea gut [41]. In both cases, biofilm formation is described as having a role in the transmission of the pathogen to the accidental host. Additionally, bacterial biofilms are considered colonization factors since they may contribute to bacterial evasion from the immune system [42–44]. Finding *Leptospira* biofilms in rats’ renal tubules may have implications in *Leptospira* survival and transmission.

We noticed a positive correlation between the intensity of CT count and biofilm formation, that is, the greater the number of colonized tubules, greater was the chance of renal biofilm formation by pathogenic *Leptospira*. If we consider the number of CT as a measure of *Leptospira* population inside the organ, this finding may indicate that biofilm formation *in vivo* by *Leptospira* is dependent on leptospiral numbers during renal colonization, suggesting the presence of quorum sensing mechanisms [45]. Moreover, our data showed that Norway rats with renal biofilm excreted ten times less leptospires in the urine than rats positive for renal infection but negative for biofilms, what we believe is a consequence of leptospires maintenance in the renal biofilm. The majority of animals presenting biofilms had a marking pattern of IHC anti-*L*. *interrogans* of partial/complete occlusion of CTs, as previously observed for naturally infected rats with no information about biofilm occurrence [11,12]. This complete pattern of renal colonization increased in more than three times the chance of renal biofilm development. Furthermore, 21 Norway rats naturally infected and with renal biofilm had agglomerated pattern of CT. Thus, it is possible to hypothesize that single leptospires infect the kidneys diffusely, coming from the circulatory system, migrate through tissue, multiply and colonize into the proximal renal tubules, where they form cell aggregates and biofilms [13].

In previous *in vitro* work developed by RISTOW et al. (2008), *Leptospira* biofilms were analysed by scanning and transmission electron microscopy [15]. The authors observed biofilms formed by a network of leptospires embedded in the extracellular matrix, although they did not use extracellular matrix specific stains. Thibeaux and collaborators (2020) analysed leptospiral charge in urine and kidneys of experimentally infected rodents, by qPCR, but did not explore structural aspects of biofilms formed *in vivo* [20]. Finally, Yamaguchi and collaborators (2018) used TEM to analyze the renal tubules of infected mice and suggested the presence of biofilm-like structures during tubular colonization by pathogenic leptospires; although they did not use specific dyes for biofilms [13].

We observed that *R*. *norvegicus* renal biofilms stained by AB in light turquoise blue but did not stain by MM. SEM using RR revealed the ultrastructure of the tubular biofilms with agglomerates of *Leptospira* involved in an acidic exopolysaccharidic matrix. Alcian blue, Mayer mucicarmine and ruthenium red are staining methods used to visualize the exopolymeric matrix. AB pH 2.5 stains alginate, acid mucopolysaccharides and sialomucins; MM stains sulfated and carboxylated mucins; and RR stains acid polysaccharides [46–48]. Those stains are commonly used to characterize extracellular matrix of bacterial biofilms, both in *in vitro* studies [28] and in *in vivo* studies [27,29,48–50]. A recent *in vitro* study showed that alginate lyase treatment did not digest the biofilm of *Leptospira*, suggesting that alginate is not a component of the exopolymeric matrix [20]. Besides, in the transcriptome study of *Leptospira biflexa* saprophytic species in mature biofilm (48 h) there was downregulation of alginate-related genes [19]. Altogether, those data indicate that the exopolysaccharidic matrix of *Leptospira* biofilm inside the renal tubules of chronically infected *R*. *norvegicu*s is composed of acid mucopolysaccharides, but not alginate.

The histopathology of infected rats is well known in the literature [9,51]. Hence, in the present work, we focused on the novelty of the histopathology of *R*. *norvegicus* naturally infected with *Leptospira* and positive for renal biofilm. Histopathological analysis revealed minimal alterations. The most frequent alterations were hyaline-goticular degeneration and chronic inflammation, both occurring in 50% of the rats. Another frequent alteration was the interstitial nephritis (discrete and moderate) that together occurred in 46% of the rats. Those results are in agreement with previous studies of naturally infected *R*. *norvegicus* [9,10,52]. In the biofilm phenotype, although bacteria are more sessile and encased in a exopolymeric matrix, it can result in some tissue alterations [53], as we observed in this study. However, since synanthropic *R*. *norvegicus* are exposed to many environmental factors, including other pathogens, it was not possible to determine if those kidney alterations were due to the presence of leptospires or their biofilms, or a consequence of the multiple factors the rats are exposed in the environment.

The prevalence of *Leptospira* in naturally infected *R*. *norvegicus* was 90%, in agreement with previous literature for Brazil and other countries (BOEY; SHIOKAWA; RAJEEV, 2019; COSTA et al., 2015b). Leptospiral shedding in urine was 1.45e+07 GEq, in accordance with the previous literature, where a range of 1.0e+05 to 1e+07 leptospires were count per mL of rat urine, by dark field microscopy [54,55]. 97% of the rat males captured and all the adult rats were positive for *Leptospira* infection. The intensity of colonization varied greatly among infected rats and was associated with demographic data, with the greatest amount of CT in males and juveniles. Male and adult *Rattus norvegicus* have a social behavior of huddling and fighting, aside from active search for food. Juveniles, on the other side, have a social play behavior in which they learn adult behavior [56]. Those juvenile rats’ behavior could augment the direct contact with contaminated environment, leading to the observed prevalence of infection.

One limitation of our study was that we were not able to directly quantify live leptospires in the rats’ kidneys and urine, but rather estimated *Leptospira* quantity using qPCR. Another limitation was the small sample of *Rattus norvegicus* with renal biofilm formation (n = 24). Thus, the prevalence here described should be considered as suggestive, instead of typical.

To date, most of the studies of *in vivo* biofilms were developed with experimentally infected hosts. Here, we demonstrated that *Leptospira interrogans* produce renal biofilm during infection in naturally infected *Rattus norvegicus* captured from an endemic site. This is an important finding on the biology of this host-adapted pathogen. *Leptospira* biofilm formation in rats’ renal tubules may have implications in bacterial survival and transmission. The biofilm phenotype in animal host reservoirs may probably impact the disease transmission cycle and should be further investigated.

## Supporting information

S1 Fig Flow diagram of the study design. (TIF)

S1 Table Raw data. (XLSX)

S2 Table Negative controls data. (PDF)
