# Supplementary figures and images for: Leptospira interrogans biofilm formation in Rattus norvegicus (Norway rats) natural reservoirs
Source: PLoS Negl Trop Dis. 2021 Sep 8;15(9):e0009736. doi: 10.1371/journal.pntd.0009736 (PMC8451993; doi:10.1371/journal.pntd.0009736)

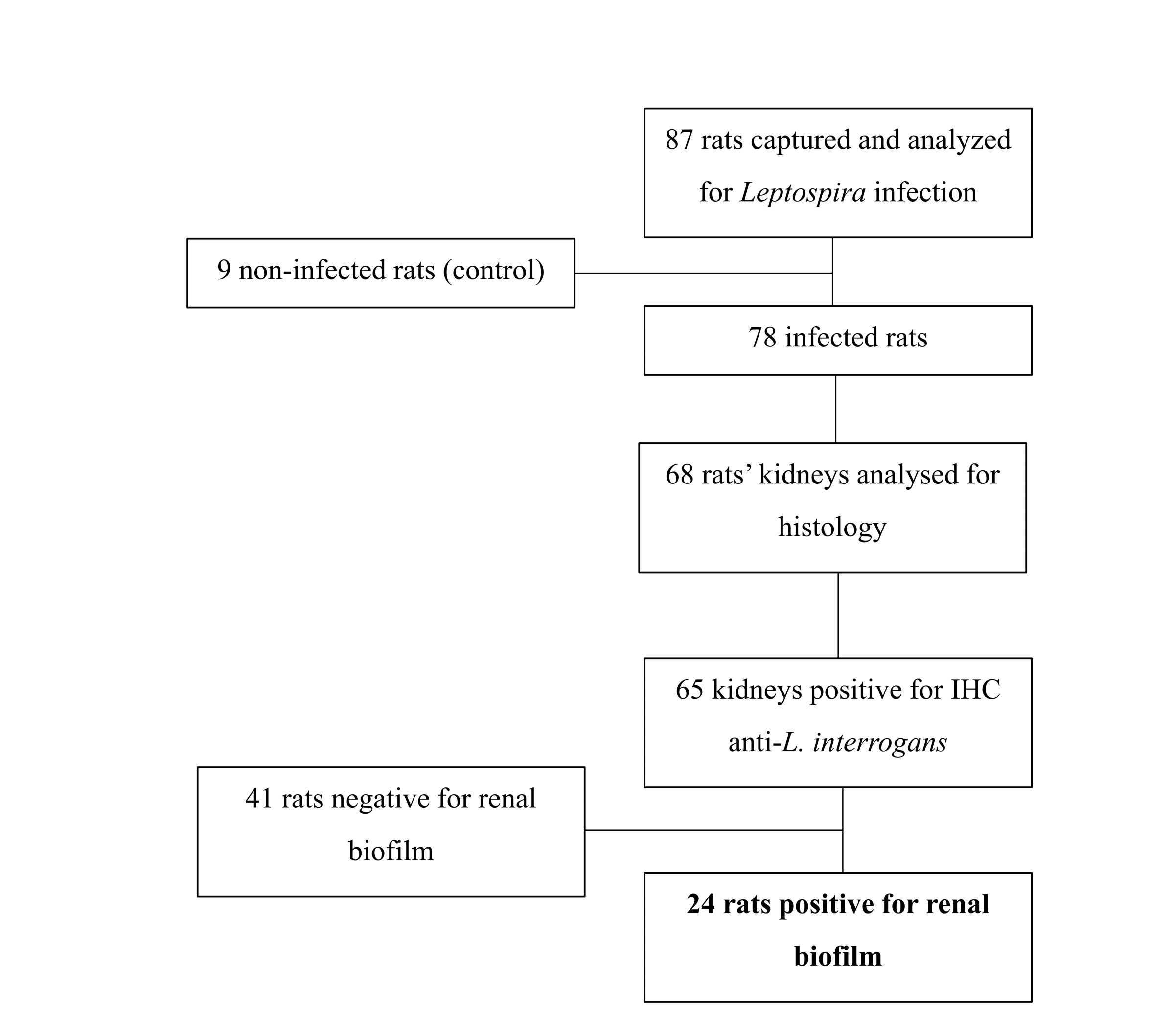

Supplement: S1 Fig — (TIF) [file pntd.0009736.s001.tif]
