# Supplementary material for: Leptospira interrogans biofilm formation in Rattus norvegicus (Norway rats) natural reservoirs
Source: PLoS Negl Trop Dis. 2021 Sep 8;15(9):e0009736. doi: 10.1371/journal.pntd.0009736 (PMC8451993; doi:10.1371/journal.pntd.0009736)
Supplement: S2 Table — (PDF) [file pntd.0009736.s003.pdf]

	Alcian Blue Stain	Mucicarmine Stain
Rats positive for *Leptospira* infection by immunohistochemistry (IHC) (n=65)	Positive (n=24)*	Negative
Rats negative for *Leptospira* infection	Negative	Negative
Dog intestine (positive control)	Positive	Positive

* Positive in co-localization.
